# The effect of folate supplementation and genotype on cardiovascular and epigenetic measures in schizophrenia subjects

**DOI:** 10.1038/npjschz.2015.46

**Published:** 2015-11-11

**Authors:** Vicki L Ellingrod, Tyler B Grove, Kyle J Burghardt, Stephan F Taylor, Gregory Dalack

**Affiliations:** 1 Department of Clinical Pharmacy, College of Pharmacy, University of Michigan, Ann Arbor, MI, USA; 2 Department of Psychiatry, University of Michigan, Ann Arbor, MI, USA; 3 Department of Psychology, University of Michigan, Ann Arbor, MI, USA; 4 Department of Pharmacy Practice, Eugene Applebaum College of Pharmacy and Health Sciences, Wayne State University, Detroit, MI, USA

## Abstract

**Background:**

Metabolic syndrome may be related to folate’s pharmacogenetically regulated metabolism and atypical antipsychotic (AAP) exposure.

**Aims:**

We examined folate supplementation on metabolic measures, endothelial functioning (Reactive Hyperemia Index (RHI)), and global methylation in AAP-treated schizophrenia subjects meeting NCEP-ATP-III-a metabolic syndrome criteria.

**Methods:**

Subjects were given 5 mg/day open label folate for 3 months. Baseline and end point measurements included RHI, body mass index, fasting metabolic laboratory measures, C-reactive protein, homocysteine, IL-6, and leptin. Subjects were genotyped for methylenetetrahydrofolate reductase (MTHFR) 677C/T and catechol-O-methyltransferase (COMT) 158 Val/Met, as well as global DNA methylation using the LUminometric Methylation Assay (LUMA).

**Results:**

Thirty-five subjects (mean age 50±9 years and 70% Caucasian) were included. At end point, RHI improved by 20% (*P*=0.02), homocysteine decreased 14% (*P*=0.006), and IL-6 decreased 13% (*P*=0.09). At baseline, 61% met endothelial dysfunction criteria (RHI<1.67), which decreased to 27% (*P*=0.0006) at end point. The MTHFR 677C/C+COMT 158Met/Met group also showed significant reduction in those meeting endothelial dysfunction (83% baseline and 16% end point (*P*=0.001)). Global methylation levels increased after supplementation (4.3%, *P*<0.0001), with subjects receiving olanzapine or clozapine experiencing greater methylation changes after folate supplementation. Folate may reduce AAP-associated metabolic risks.

**Conclusions:**

We report significant reductions in the number of subjects meeting endothelial dysfunction. Given that all subjects met metabolic syndrome criteria, this may prove as a useful avenue to reducing cardiovascular disease risk. MTHFR and COMT genotypes may affect response and underlying changes in DNA methylation may help to explain the mechanistic underpinnings of these findings.

## Introduction

Within the general population the incidence of cardiovascular disease is on the rise.^1^ However, for patients diagnosed with a serious mental illness, such as schizophrenia, the incidence of metabolic syndrome is more than double that is seen in the general population.^2^ Similar between both of these populations is the fact that the occurrence of metabolic syndrome is associated with significant cardiovascular disease (CVD) above the contribution of each individual component.^3^ Recent work has shown that up to 30 years of life are potentially lost for those diagnosed with a serious mental illness compared with the general population,^4^ with the majority of this loss due to cardiovascular disease. Thus, finding a way to prevent or mitigate this risk is critical as we work to improve outcomes associated with schizophrenia.

The role of folic acid, which is a water soluble B-vitamin involved in the synthesis, repair, and methylation of DNA, is garnering much attention within the mental health field.^5^ For individuals diagnosed with schizophrenia and treated with an antipsychotic, work previously done by our group and others suggest a relationship between the methylenetetrahydrofolate reductase (*MTHFR*) 677C/T variant as well as the catechol-*O*-methyl transferase (*COMT*) 158 Val/Met variant and metabolic syndrome risk.^6,7^ Furthermore, our group has found associations between folate, one carbon metabolism, DNA methylation and atypical antipsychotic (AAP) metabolic side-effect risk.^8,9^ As part of these investigations we found a significant relationship between a surrogate maker of global methylation (Long Interspersed Nucleotide Element 1 methylation), and an interaction between MTHFR and gender, after controlling for serum folate (*P*=0.008). Overall, females with the *MTHFR* 677TT genotype had the lowest methylation (56%) compared with the other male and female genotype groups (75%). In addition, our group reported gene-specific methylation differences related to *COMT* that suggest that *COMT* promoter region methylation is largely influenced by *COMT* genotype and that physical activity has a significant role in epigenetic modulation of *COMT* in the schizophrenia population. Therefore these two analyses are the basis for gaining a better understanding of the role of folate in the attenuation of metabolic complications seen with AAP use.

**Figure 1:**
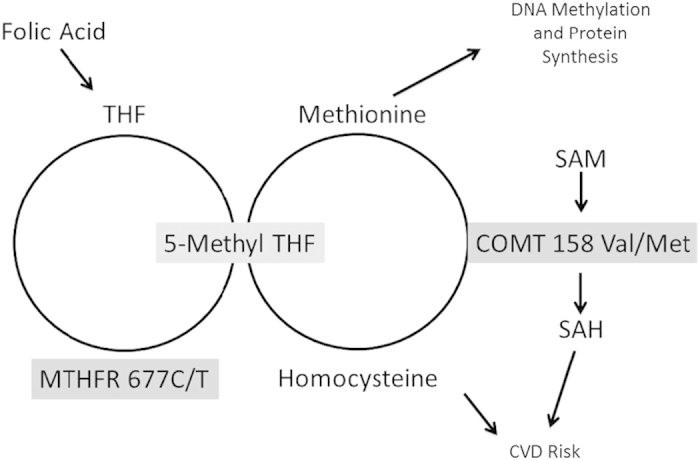
Schematic representation of intersection of one carbon metabolism and methionine metabolism on CVD risk and DNA methylation and protein synthesis. COMT, catechol-*O*-methyltransferase; CVD, cardiovascular disease; MTHFR, methylene tetrahydrofolate reductase; SAH, *S*-adenosyl homocysteine; SAM, *S*-adenosyl methionine; THF, tetrahydrofolate.

As illustrated in Figure 1, *MTHFR* is involved in facilitating formation of the active form of folic acid (5-methyl folate) through one carbon metabolism, of which homocysteine is a byproduct. Although one carbon metabolism cycle is centered on folate and is also responsible for formation of the methyl group needed for epigenetic regulation and protein synthesis.^10^ Individuals with the *MTHFR* T allele have a 30% reduction in folate metabolism for each allele, and as such this variant has been associated with hyperhomocystenemia. Within the general population, homozygosity for the 677T allele occurs in ~12% of the general population; however, evidence suggests that the percentage is higher among patients with schizophrenia.^11^

Similar to the one carbon metabolism system, *COMT* is an enzyme that is primarily responsible for catecholamine metabolism that may also affect CVD risk, but through a different mechanism. As a methyltransferase, COMT introduces a methyl group to catcholamines, which is donated by *S*-adenosyl methionine (SAM). This methyl donation results in the formation of *S*-adenosyl homocysteine (SAH), which also has been attributed to cardiovascular risk and epigenetic dysregulation.^12,13^ For *COMT*, presence of the 158 Met allele results in a thermolabile protein affecting activity compared with the 158Val variant. Those with the Val/Val genotype have 30–50% greater activity than those with the Met/Met genotype,^14^ and this genotype also appears to potentially augment folic acid and one carbon metabolism, resulting in hyperhomocysteinemia. Therefore, given their differing mechanisms of action and complementary effects on homocysteine production, both the *MTHFR* 677T and *COMT* Val alleles may potentially increase the risk for CVD, which makes both pertinent to schizophrenia and AAP-associated metabolic risks.

Thus, given our previous work showing a greater metabolic syndrome risk for those carrying a *MTHFR* T or *COMT* Val allele, we sought to determine if folate supplementation (5 mg of folic acid given for 3 months) could attenuate some of the metabolic consequences seen with antipsychotic use in patients diagnosed with schizophrenia. In terms of one carbon metabolism, we hypothesized that driving that cycle with increased folate could increase methyl donation needed for DNA methylation. To test this hypothesis, we examined global methylation within this cohort of patients for predicted increases. Previous work on neural tube defects, associated with the *MTHFR* 677T allele has shown that higher dose folic acid supplementation may help to overcome the pharmacogenetic reduction in the formation of the active form of folic acid (5 methyl folate) that is necessary as part of one carbon metabolism.^15^ Thus, we hypothesized that folate administration would result in a slight reduction in metabolic measures with either the *MTHFR* 677T allele or *COMT* 158 Val allele would show the greatest improvements, given that they would have stronger responses to the pharmacologically induced drive. Lastly, we predicted an improvement in endothelial functioning (used as a surrogate marker for overall CVD).

## Materials and Methods

### Subject population

Subjects included in this study were recruited from our cross-sectional phase I study that initially screened for metabolic syndrome.^16^ As part of this folate supplementation trial subjects had to meet metabolic syndrome criteria^17^ defined by any three of the following: (1) abdominal obesity characterized by waist circumference of ⩾40 inches for men or ⩾35 inches for women, (2) triglycerides ⩾150 mg/dl, (3) HDL cholesterol <40 mg/dl for men and <50 mg/dl for women, (4) blood pressure ⩾130/85 mm Hg or treatment for hypertension, or (5) fasting glucose ⩾100 mg/dl or treatment of diabetes.^18^ In addition, the subject’s vitamin B12 levels needed to be within normal laboratory limits. The exclusion criteria included (1) unable to give informed consent (assessed using a short questionnaire asking key questions about the study), (2) active substance abuse disorder and (3) medication changes in the past 3 months. All subjects gave written, informed consent to participate in the protocol, which was approved by the University of Michigan Medical School Institutional Review Board (IRBMED) and is also registered with clinicaltrials.gov (NCT00815854).

### Assessments

Subjects meeting study inclusion/exclusion criteria underwent informed consent followed by a clinical interview including, an assessment of current and past medication history, smoking status (including current and past use), and ethanol intake. Subjects also underwent an interview to assess psychiatric symptoms using the Brief Psychiatric Rating Scale (BPRS),^19^ Scale for the Assessment of Negative Symptoms (SANS),^20^ Beck Depression Inventory (BDI),^21^ and Psychological Stress Index (PSI).^22^ These assessments were completed at each visit for 3 months with the same raters being used for all subjects (Grove and Taylor) in an effort to reduce inter-rater variability.

Before their baseline and endpoint study visit, subjects fasted for at least 8 h. The visits were also timed to take place between 0800 hours and noon, so that they occurred within 2 h of the subject’s usually waking time based on appointment availability. Vital signs, as well as height, weight, and hip and waist circumference was measured for each subject and Body Mass Index (BMI, kg/m^2^) and hip/waist ratio was calculated. Blood was also drawn at the baseline and endpoint visit for pharmacogenetic and epigenetic analysis in addition to the following fasting laboratory assessments, which were conducted by the Michigan Diabetes Research and Treatment Center (MDRTC) core laboratory: folate, vitamin B12, homocysteine, glucose, insulin, hemoglobin A1c, lipids, leptin, TNF-a, IL-6, and adiponectin. Each subject’s average physical activity level was calculated by using the Total Activity Measure 2 (TAM2), which measures total or moderate intensity physical activity by asking participants for the total time spent in activity at different activity levels per week.^23^ The amount of physical activity is calculated by conversion to Metabolic Task Equivalents per minute (MET/min). Side effects from folate administration was measured using the Udvalg for Kliniske Undersøgelser (UKU) Side Effect scale administered monthly.^24^

### Endothelial function assessment

Arteriole endothelial-dependent vasodilatation (aka endothelial functioning) was assessed using the EndoPAT 2000 device (Itamar Medical, Caesarea, Israel), which has been validated and described in previous studies as a non-invasive method using peripheral arterial tonometry (Reactive Hyperemia Index, RHI) signals.^25–31^ This commercially available FDA approved device provides a clinically useful metric of endothelial-dependent vasodilatation.^25,26^ Our previous reports describe this assessment in greater detail, but briefly, two specifically designed finger probes are placed on the subject’s index finger of each hand. They are then inflated through the use of pneumatic tubes connected to an inflating device controlled by a computer algorithm. Subjects remain motionless for 5 min during the baseline measurement. A blood pressure cuff located on the subject’s non-dominant arm is inflated to occlude the brachial artery and subjects remain motionless during this time while occlusion is confirmed by a reduction of the RHI tracing to zero. After 5 min, the blood pressure cuff is rapidly deflated and the subject is instructed to stay motionless for another 5 min for the final assessment.

This assessment results in an RHI value that is automatically generated by the computer algorithm and calculated as the ratio of the PAT signal after blood pressure cuff release compared with the baseline evaluation. The final RHI output is normalized for baseline signal and indexed to the contralateral arm. Subjects with an RHI index of <1.67 meet criteria for endothelial dysfunction as this value yields 80% sensitivity and 85% specificity to identify endothelial dysfunction.^26,29^

### Folate supplementation

Subjects received a prescription for 5 mg of folic acid daily, which was filled and given to the subject by the research coordinator. Due to the prescription availability of folate, subjects took a single dose of five 1 mg folic acid tablets. Adherence was measured through pill counts done at each visit.

### *MTHFR* and *COMT* genotyping

A whole blood sample was obtained at each subject’s baseline and endpoint visit from which genomic DNA was isolated using salt precipitation. Genotyping was done with Pyrosequencing Technology for the *MTHFR* 677C/T (rs1801133) and *COMT* Val158Met (rs4680) variants. Assay conditions are available upon request. Genotype calls were made blinded to participant assessments. Ambiguous calls were repeated with a consensus assessment of genotypes. Call rates were 99% for these assays.

### Epigenetic analysis

An aliquot of DNA sample was also used for an assessment of global methylation using the LUminometric Methylation Assay (LUMA)^32^ on samples obtained at baseline and endpoint allowing us to calculate percent change in methylation. Conditions for this assay are also available upon request.

### Statistical analysis

Differences in primary outcomes and socio-demographic variables were determined with one-way analysis of variance (ANOVA) for normally distributed variables. Antipsychotic classification was based on weight gain liability with subjects receiving olanzapine, clozapine, quetiapine, risperidone, or paliperidone, being classified as receiving a weight gain associated AAP. Subjects receiving ziprasidone and aripiprazole were classified as receiving a lower weight gain–associated AAP due to their lower metabolic risk.^33,34^ Hardy–Weinberg equilibrium for genotypes was examined using Haploview.^35^ Due to the small sample size, subjects were categorized by allele status resulting in four groups consisting of the *MTHFR* CC genotype and T-allele carriers and *COMT* Met/Met genotypes and Val allele carriers. Differences between baseline and endpoint were determined for measured variables using a matched pairs model in JMP 9 statistical software where the subject’s baseline visit serves as the control. In addition, we calculated percent change in all continuous variables (laboratory and rating scales) to determine the relationship between these variables and demographic differences (i.e., race, gender, sex, smoking, AAP use) and genotype (*MTHFR* 677 C/T and *COMT* 158 Val/Met) using a logistic regression model. Changes in methylation were analyzed using repeated measures ANOVA and multivariate analysis of variance (MANOVA) based on the LUMA results measured from DNA obtained at baseline and study endpoint. A *P* value <0.05 was considered statistically significant.

## Results

A total of 35 patients were included in this analysis. Of these subjects, 23 were male (66%) and 24 classified themselves as Caucasian (70%), followed by 9 African American (23%) and 2 Other (3%). The mean age of subjects was 50.5±8.9 years and the mean BMI at baseline was 37.9±9.48 kg/m^2^ (95% CI: 33.7– 42.1). Seventy-six percent of subjects (*n*=27) were receiving AAPs and subject met an average of 4.2±1.5 of the metabolic syndrome criteria (95% CI: 3.6–4.7). In addition, the mean RHI at baseline was 1.76±0.56 and 67% of subjects meet criteria for endothelial dysfunction (RHI <1.67). All subjects were psychiatrically stable at baseline with a mean BPRS of 30±5, mean SANS of 5.5±3.2, mean BDI of 12.3±8.9, and mean PSI of 2.24±0.53. Of the 35 subjects, a total of 2 withdrew from the study. One subject withdrew due to the need to start metformin (as medication changes were an exclusion) and the other subject was withdrawn due to medication non-compliance with the folate. Table 1 is a description of additional subject demographics for all 35 subjects. In addition, all genotypes were in Hardy–Weinberg distribution (*P*>0.05).

Overall folate administration was very uneventful with 27 of the 35 subjects reporting no adverse events using the Udvalg for Kliniske Undersøgelser (UKU) Side Effect scale, which was administered monthly. This standardized scale rates adverse events on a scale of 0–3 in several domains. Of the eight subjects who did have side effects, none of them ranked any side effects >2 and the most common side effects were gastrointestinal in nature. Unfortunately, the incidence of these adverse drug reactions is only noted in the package insert as common with no exact percentage given. Table 2 lists the prevalence of adverse drug reactions by study visit. As previously mentioned, one subject was withdrawn due to medication non-compliance. In looking at the remaining subjects, medication compliance was estimated to be at 90% based on the amount of unused medication returned to the study team.

**Figure 2:**
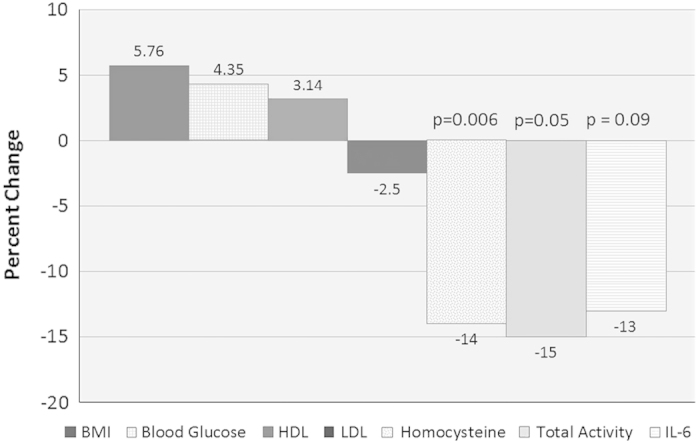
Percent change in main metabolic and lifestyle measures after folate administration for 3 months.

After 3 months of folate administration, the mean RHI increased by 14±30% (*t*=2.05, *P*=0.04) indicating better functioning. At baseline, 67% of subjects met endothelial dysfunction criteria (RHI<1.67), which decreased to 27% (*χ* ^2^=−3.84, *P*=0.0006) at endpoint. As outlined in Figure 2, mean homocysteine levels decreased by 14% (*P*=0.006) and IL-6 decreased 13% (*P*=0.09). We also found that subjects exercised less during the study, with the TAM2 scores decreasing 15% (*P*=0.05). We did not see any significance differences in total cholesterol, triglycerides, blood pressure, blood glucose, insulin resistance, waist circumference or BMI after folate administration (*P* value >0.1 for all).

**Figure 3:**
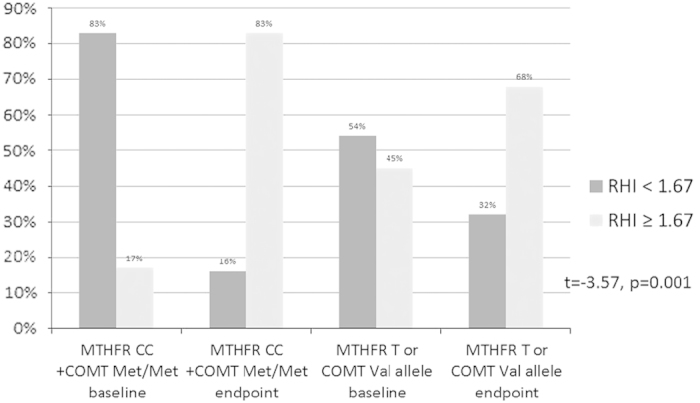
Differences in percent of patients meeting endothelial dysfunction criteria before and after folate supplementation stratified by MTHFR/COMT genotype.

Overall, the percent change in RHI was not related to age (*P*=0.21), race (*P*=0.15), sex (*P*=0.78), cigarette smoking (*P*=0.83), or AAP use (*P*=0.12). In looking at the effect of the two genotypes separately, no significant relationships were found; however, clinically significant differences in mean RHI improvement were found for both that represent a Cohen’s effect size of 0.4. Overall subjects with the *MTHFR* 677T allele had a mean RHI improvement of 19.4±33.1% compared with the *MTHFR* CC genotype group with 6.7±7% (*P*=0.29). Similarly for the *COMT* Val allele carriers, the mean RHI improvement was 23.2±42.3 vs. 9.8±21.7 for the Met/Met genotype group (*P*=30). For the combined effects of these genotypes, the *MTHFR* 677C/C+*COMT* 158Met/Met genotype subjects had a 44% RHI improvement compared with a 10% improvement for *MTHFR* 677T/*COMT* Val allele carriers; however, this just showed a statistical trend (F(3,26)=3.22,*P*=0.06). Those with the *MTHFR* 677C/C+*COMT* 158Met/Met genotypes had a significant reduction in percent of subjects meeting endothelial dysfunction criteria (83% at baseline and 16% at endpoint), compared with the *MTHFR* T/*COMT* Val allele carriers (54% at baseline and 31% at endpoint; *t*=−3.57, df=27, *P*=0.001) as shown in Figure 3.

In looking at the psychiatric effects of folate, we saw significant improvement in psychological stress measured using the PSI (*t*=−3.06, *P*=0.02), as well as depressive symptoms measured using the BDI (*t*=−2.75, *P*=0.04), however, due to the open label design of this study, these results need to be very cautiously interpreted. No significant change in primary psychiatric symptomatology was noted using the BPRS (*P*=0.14) or SANS (*P*=0.37).

### Epigenetic analysis

Overall, the percent global methylation as measured by LUMA increased after folate supplementation (74.0% before vs. 78.3% after, *t*=4.50, *P*<0.0001). This percent change in global methylation did not correlate with percent change in folate levels (*P*=0.9). A MANOVA was conducted using percent change in global methylation as the response variable and gender, antipsychotic type, baseline BMI, and baseline folate levels as explanatory variables. The explanatory variables were chosen due to their previously shown effects on global methylation levels.^8,36,37^ This model was significant primarily due to the effect of antipsychotic type (*P*=0.0066) on global methylation (F(5,21)=3.16,*P*=0.035). The effect of gender, baseline BMI, and baseline folate on change in global methylation were not significant (*P*=0.2, 0.9, and 0.5, respectively). As outlined in Table 3, subjects treated with olanzapine and clozapine had significantly higher increases in global methylation (+5.43%) as compared with subjects treated with any other antipsychotic type (+2.22%) (F(35,1)=4.78, *P*=0.04). Finally, we investigated the effect of our genotype groups on global methylation after folate supplementation. Neither the *MTHFR* 677C/T, *COMT* 158Val/Met or the combined effects of the genotypes significantly influenced changes in global methylation (both *P*>0.3).

## Discussion

Overall as part of this pilot investigation, we report that high-dose folate supplementation may be effective in attenuating some of the cardiovascular effects seen in those using atypical antipsychotics, while having little impact on core schizophrenia symptoms, and a modest positive effect on depressive symptoms and psychological stress. In addition, administration of the folate supplementation was well tolerated by our study subjects. Most importantly folate supplementation may help reduce the number of subjects meeting endothelial dysfunction criteria, which is remarkable given that all subjects included in this study met metabolic syndrome criteria. This change in endothelial functioning resulted in amelioration of a medical complication for subjects, which may lead to reduced CVD risk. We purposely chose to examine the effects of folate on the non-invasive measurement of endothelial functioning as a biomarker, as this non-invasive measurement allows for an overall CVD risk estimation compared with focusing on solely weight loss and/or glucose regulation. The endothelium is paramount to vascular biology and is responsible for the production of nitrous oxide, which has anti-inflammatory effects and therefore helps inhibit atherosclerosis. Dysfunction of this vital organ contributes to CVD development. The hyperhomocysteinemia resulting from poor folate metabolism related to *MTHFR/COMT* or poor folate intake directly damages endothelial cells and impairs nitrous oxide release, reducing endothelial functioning.^38,39^ Thus, supplementing the folic acid cycle with 5 mg of folate for 3 months may work to overcome endothelial damage and may help to improve overall endothelial functioning, potentially through an anti-inflammatory process given our reduction in measured cytokines, and relative lack of change in metabolic measures.

In addition, we also found that 3 months of folate supplementation has a significant effect on global methylation levels in schizophrenia subjects taking antipsychotics and having metabolic syndrome. On the basis of involvement of the folate cycle in antipsychotic metabolic side effects and the production of methyl donors, our main findings confirm current epigenetic hypotheses by showing an overall increase in global methylation after folate supplementation that was significantly influenced by antipsychotic type. Subjects taking the most metabolically adverse antipsychotics, olanzapine and clozapine, had the lowest methylation levels at baseline along with the largest increases in methylation levels after folate supplementation (*P*=0.04, Table 3). Furthermore, when looking at medication differences by the changes in global methylation were larger in females (Table 3). This result is in line with results we have previously reported related to gender differences in global methylation levels^8^ and other studies showing antipsychotic effect on methylation levels.^40^ Together, these findings may indicate that subjects treated with olanzapine and clozapine are in a global hypomethylated state and are uniquely suited for folate supplementation in order to bring the global methylation values back to a “normal” state and that the effect of folate supplementation on DNA methylation may be more pronounced in females.

Currently very little work has been done in regards to epigenetic changes related to folate administration within the schizophrenia population. Therefore, our results may aid in the understanding of how folate supplementation influences antipsychotic-associated metabolic side effects, as well as the potential for global methylation levels serving as a potential biomarker for folate supplementation in schizophrenia. Further work is needed to correlate global methylation changes to metabolic outcomes of interest including insulin resistance, dyslipidemia and endothelial dysfunction. Furthermore, targeted epigenetic approaches need to be taken in order to investigate gene-specific methylation changes and their association with antipsychotic-metabolic side effects, if any.

In comparing our significant results in overall endothelial functioning improvement, it is somewhat hard to make direct comparisons as AAP metabolic interventions have been limited to metformin and topiramate, focusing on weight loss and glucose regulation, with no overall global CVD assessment. While metformin is FDA approved for glucose control, topiramate is an anticonvulsive. Both carry adverse risks and costs; and their efficacy when used for this purpose has been questioned,^41–44^ although both are being used routinely in clinical care. In looking at the adverse events seen with folate administration, very few issues were determined through our routine monitoring, with the most common side effect being constipation that occurred in 14% of subjects.

As part of this investigation we also routinely monitored for psychiatric symptomatology and found very little effect of our folate intervention. Overall subjects remained psychiatrically stable, while experiencing some non-significant improvements in depressive symptoms as well as psychological stress (data not shown). In looking at the literature, only one other investigation has examined the role of supplemental folate on negative symptom presentation in patients with schizophrenia.^11^ The study included 32 patients who were randomized to either receive 2 mg/day of folate or placebo. The primary study assessment was the SANS in Schizophrenia and the primary aim was to determine the effect of 12 weeks of folate supplementation on this measure. Overall, this investigation showed that 2 mg/day of folate supplementation was more likely to improve negative symptoms for patients with at least one copy of the T allele (*P*=0.01).^11^

While the results of this trial are interesting there are a few limitations that need to be acknowledged. First, this was an open label trial of folate supplementation that included a small number of subjects. Current work being done by our group is now examining the metabolic effects of folate using a randomized placebo-controlled design. In addition, the inflammatory measures used for this investigation, consisted of two measures, therefore our ongoing study is accessing inflammation using a standard inflammatory panel. Lastly, our epigenetic measurement only accessed global methylation in peripheral blood given our small sample size, and therefore future investigations will include gene-specific and tissue-specific methylation differences to determine the mechanistic underpinning of folate’s effects.

## Conclusion

Overall folate supplementation may reduce AAP-associated metabolic risks and we report significant reductions in the number of subjects meeting endothelial dysfunction. This is remarkable given that ALL subjects met metabolic syndrome criteria. This may prove as a useful avenue to reducing CVD risk. Those with the *MTHFR* T or *COMT* Val alleles may not benefit from folate, but this needs further follow-up. Finally, further work is needed to correlate global methylation changes to metabolic outcomes of interest including insulin resistance, dyslipidemia, and endothelial dysfunction. Targeted epigenetic approaches need to be pursued in order to investigate gene-specific methylation changes and their association with antipsychotic-metabolic side effects, if any.

## Figures and Tables

**Table 1:** Subject baseline demographics

*Demographic variable*	N*=35 subjects*
Mean age±s.d (years)	50.5±8.94 years
Race (%)	70% Caucasian, 26% African American and 3% Other
Male/female (%)	66%/33%
Receiving an AAP (%)	76%
BMI (±s.d.) kg/mm^2^	37.9±9.48
Blood pressure±s.d (mm Hg)	136/76±20/11
% Cigarette smoker	58%
No. of metabolic syndrome criteria met±s.d.	4.2±1.5 (95% CI: 3.6–4.7)
Baseline RHI±s.d	1.8±0.56
*MTHFR* genotype groups (*N* and %)	T allele (*n*=15, 43%) C/C genotype (*n*=20, 57%)
*COMT* genotype groups (*N* and %)	Val allele (*n*=23, 66%) Met/Met genotype (*n*=12, 34%)

Abbreviations: *COMT*, catechol-*O*-methyltransferase; *MTHFR*, methylenetetrahydrofolate reductase; RHI, Reactive Hyperemia Index.

**Table 2:** Side effects reported using the UKU side effect scale

*ADR % (total number)/visit number*	*1 month*	*2 month*	*3 month*
Reduced salivation (dryness of mouth)	3% (*n*=1)	3% (*n*=1)	0% (*n*=0)
Nausea/vomiting	3% (*n*=1)	6% (*n*=2)	3% (*n*=1)
Diarrhea	3% (*n*=1)	6% (*n*=2)	6% (*n*=2)
Constipation	14% (*n*=5)	6% (*n*=2)	14% (*n*=5)
Polyuria/polydipsia	8.5% (*n*=3)	8.5% (*n*=3)	6% (*n*=2)

Abbreviation: ADR, adverse drug reaction.

**Table 3:** Changes in global methylation based on antipsychotic group

*Antipsychotic group for entire pilot sample*	*Baseline % global methylation*	*Endpoint % global methylation*	*Change in % global methylation*
Olanzapine and clozapine	74.0	79.5	5.44
All other antipsychotics	75.2	77.5	2.23

*Antipsychotic group based on gender*	*Males*	*Females*	*Males*	*Females*	*Males*	*Females*
Olanzapine and clozapine	73.6	75.8	78.4	83.5	4.80	7.65
All other antipsychotics	76.0	74.2	77.8	77.0	1.85	2.74

This table shows the changes in % global methylation based on antipsychotic type. Antipsychotic groups were arranged according to known propensities for causing metabolic side effect. Olanzapine and clozapine are the most metabolically adverse and were compared with all other atypical antipsychotics in the study. The olanzapine and clozapine group had the lowest % global methylation values at baseline and they had the largest increase after supplementation. The changes in global methylation were larger in females.
